# Lessons Learned: Why Motivational Interviewing Should Be Adapted to Socio-Cultural Contexts

**DOI:** 10.3390/healthcare14081059

**Published:** 2026-04-16

**Authors:** Christine Kirby, Julie A. Baldwin, Kristan Elwell, Michelle Anne Parsons

**Affiliations:** 1 Center for Community Health and Engaged Research, Northern Arizona University, Flagstaff, AZ 86011, USA; 2 Department of Health Sciences, Northern Arizona University, Flagstaff, AZ 86011, USA; 3 Department of Anthropology, Northern Arizona University, Flagstaff, AZ 86011, USA

**Keywords:** community health, cultural tailoring, oral health interventions, diverse communities, motivational interviewing, interdisciplinary health research

## Abstract

Background: The literature shows inconclusive results from utilizing motivational interviewing (MI) in indigenous populations to address early childhood caries (ECC). Great Beginnings for Healthy Native Smiles (GBHNS) (NIDCR U01DE028508), a community focused oral health (OH) intervention, was utilized alongside adapted MI techniques to promote OH care and education at home. Methods: The intervention was conducted by local Community Health Representatives (CHRs) from the two partnered indigenous communities. Reflecting on the years-long MI training and CHRs’ concerns, GBHNS conducted post-intervention semi-structured interviews with all MI staff regarding their experiences with MI. This paper uses participant observation, semi-structured interviewing, and inductive and deductive qualitative coding and analysis. Results: Thematic analysis was used to explore lessons learned and future research recommendations for interventions considering the use of MI. Generally considered a person-centered approach, MI reinforces Western psychological frameworks and practices which may disrupt local communicative practices and values. Conclusions: Specifically, interdisciplinary pre-intervention community assessments are recommended to ensure acceptability, relevance and appropriateness through attention to local communicative practices.

## 1. Introduction

Tailoring public health interventions to specific populations has been shown to improve their effectiveness and cultural relevance [1,2,3,4,5,6,7,8,9]. One critical yet often overlooked dimension of tailoring is the language through which interventions are delivered. Research assessing cultural tailoring to various degrees often focuses on health communication as “messaging” or text-based information tailored to populations in various ways [10]. While this is valuable work and an insight into salient messaging, some public health interventions employ “talk based” methods like motivational interviewing (MI) to impact behavior change. Research in linguistic anthropology and sociolinguistics demonstrates that language is not merely a vehicle for information but a social action that constructs identities, relationships, and reproduces power relations. Through linguistic indexing, language can signify inclusion or exclusion, reinforcing or challenging social boundaries [11,12,13,14,15].

Attention to how populations conceptualize, use, and reproduce language offers valuable insight for public health communication and intervention design [10,15,16,17]. This is especially crucial in interventions that rely heavily on interpersonal communication, such as motivational interviewing or counseling, and are grounded in Western psychological frameworks. In this manuscript, we examine one such intervention that employed MI within a Tribal public health context. During the MI training and implementation phases, staff encountered a range of communicative challenges and cultural disconnects. The findings suggest that while MI holds promise, its effectiveness depends on adaptations that align with locally centered knowledge, values, and communicative practices. Briggs shows us that metacommunication, or “language describing or evaluating communicative events or processes” [16], is key in the procedure of understanding what communicative practices are appropriate in various events and regarding various fields of inquiry. This, in turn, requires public health researchers to critically reflect on their own communicative practices and engage deeply with those of the populations they serve when tailoring interventions.

The aim of this paper is to examine how language use and interpersonal communication shape the implementation and effectiveness of public health interventions, drawing primarily on post-intervention interviews with motivational interviewing (MI) specialists and Community Health Representatives (CHRs) involved in the GBHNS intervention. CHRs reflected on the challenges and rewards of learning and applying the adapted MI approach, noting that while some communicative strategies aligned with their communities’ practices, others did not. We begin by reviewing the literature on (1) the use of motivational interviewing in Indigenous communities, including studies focused on reducing early childhood caries (ECC). We also (2) engage linguistic anthropology research that critiques the practice of MI and highlights the embeddedness of language in social interaction. This review is followed by feedback from GBHNS MI staff and consultants, who reflect on the benefits and cultural disconnects they encountered when using MI. Although MI is often presented as a universally applicable person-centered approach across speech communities [18], evidence of outcomes and implementation challenges in Indigenous contexts suggests the need for further investigation [19,20,21,22,23,24,25,26,27,28]. Drawing on concerns voiced by Indigenous practitioners involved in the GBHNS intervention, this paper illustrates how MI may misalign with local communicative practices, thus de-centering the population it is serving. We conclude by recommending that public health researchers use interdisciplinary methods to assess community-specific communicative practices prior to or alongside culturally tailoring interventions.

During the creation, collaboration, implementation and analysis of GBHNS, the 2012 Miller and Rollnick resource was utilized for foundational documents, training, and analysis. After the intervention was completed, a 2023 version has been published with minor changes to the definitions of functions within MI and how MI can be altered to be utilized in cultural contexts [19]. The authors find the 2023 representation of cultural adaptations to MI to still miss the foundations of the issues we present here: not all cultures are monoliths; concepts of culture must recognize the dynamic nature of culture. By extension, the act of cultural adaptation is a meaning making process that must also represent local linguistic practices. As stated by Abu Lughod “Every view is a view from somewhere and every act of speaking is speaking from somewhere [20].” Various researchers have attempted to adapt MI to cultural contexts [21,22,23,24]. The research team supports the recommendations from Venner [24], which still are lacking in the most recent attempt of incorporating cultural considerations to MI [19]. Venner recommends that MI must address power imbalances, acknowledge the limitations of Western methods in capturing Indigenous knowledge and perspectives, ensure mutually supportive partnerships, recognize and uphold Indigenous ways of knowing, being, and doing, prioritize Indigenous self-determination, and prioritize Indigenous-led approaches. From Jamieson [23], we also support capturing contextual relevance and nuance; in this case, the team would like to especially highlight the contextual relevance and nuance of speech communities. In this work, we have come to realize that many cultural tailoring attempts of MI neglect linguistic formative assessments and observation of local linguistic practices in order to prioritize a salient practice of MI with local values.

## 2. Background

### 2.1. Motivational Interviewing and Indigenous ECC Interventions

Stemming from the client-centered Rogerian movement in psychology, motivational interviewing strives to enact behavior change through conversation [29]. Designated as an evidence-based practice in the early 1980s by the National Registry of Evidence-Based Practices and Programs (NREPP, now part of the Pew Foundation), MI quickly spread from therapy-centered practices to researchers. Originally developed to help clients articulate their own motivations for behavior change, motivational interviewing has since been adopted as both a research method and a practical tool across a range of fields, including dentistry, medicine, corrections, nutrition, and social work. For example, public health interventions have adopted the evidence-based practice, utilizing it to treat addiction, smoking cessation, nutrition and fitness, oral health, and other public health-related areas [2,21,22,24,30,31]. 

MI practitioners tactfully direct clients through conversation. It is through this conversational direction that a type of client talk is encouraged while another is left to fall away; ‘change talk’ over ‘sustain talk’ respectively. Change talk is a person’s own talk that favors change and sustain talk is one’s talk which focuses on maintaining current practices or resistance to change [18,32]. Ultimately, MI is focused on working through client ambivalence [29]. By listening, guiding, and encouraging clients’ change talk, MI practitioners work toward “strengthening a person’s own motivation and commitment to change” [18,32]. Responding to participants in a way to elicit “change talk” and allow “sustain talk” to fall away from the focus of conversation is key [29]. Nudging clients away from ambivalence and toward change talk is the overall goal.

In addition to Cultivating Change Talk and Softening Sustain Talk, MI also values partnership and empathy. Though not the central training materials for GBHNS, a culturally adapted version of MI highlights collaboration mutual respect and equality [32,33]. Partnership is defined as “the extent to which the clinician conveys an understanding that expertise and wisdom about change reside mostly within the client” [34], while empathy is defined as “the extent to which the clinician understands or makes an effort to grasp the client’s perspective and experience” [34] (p. 11, see also Miller & Rollnick, 2023; Miller & Moyers, 2021). Knowing when and how to respond to clients in the spirit of MI takes years of dedicated and consistent practice, testing, and requisite fees [25]. Measures or definitions of what clients experience as empathy or partnership are not scored or recorded as part of the measure.

A validated scoring system is used to measure MI proficiency. Accepted as the test of MI competence and proficiency, the Motivational Interviewing Treatment Integrity Coding Manual (MITI) assists users in reviewing an auditory 20 min segment of an MI session and scoring the appropriate indicators and phenomena of MI [34]. The MITI scoring system produces global ratings on a 5-point Likert scale across four dimensions: Cultivating Change Talk, Softening Sustain Talk, Partnership, and Empathy. In addition to these global scores, the MITI includes behavior counts—independent tallies of specific practitioner behaviors observed during the 20 min segment. These behaviors include Giving Information, Persuade, Persuade with Permission, Question, Simple Reflection, Complex Reflection, Affirm, Seeking Collaboration, Emphasizing Autonomy, and Confront. More about each of these can be found in the MITI Manual [34]. Later in this manuscript we will define and go into more detail about complex reflections and affirmations. From these behavior counts, ratios such as the reflection-to-question ratio and the proportion of MI-adherent behaviors are calculated. MI proficiency is then assessed based on a combination of global scores and behavior-based metrics. The MITI is the gold standard for evaluating the fidelity of MI delivery in both interventions in clinical practice and research.

Studies have measured the impact of MI on oral health outcomes in Indigenous communities with mixed results [19,23,24,25,26]. In a metanalysis of MI interventions focusing on ECC, the authors note a lack of oral health related standardized protocols for MI counseling which makes drawing conclusions systematically quite difficult; however, with the three articles contributing to the metanalysis, MI was as effective as oral health education in controlling ECC [27,28]. In a systematic review, seven studies were analyzed and, while MI was successful in motivating patients to get to appointments and use fluoride along with overall knowledge increase, other outcomes were inconclusive [28]. The results from these analyses illustrate and recommend the need for more research and better designed and reported interventions to assess the impact of MI on early childhood caries. A recent study involving Indigenous children and their parents in Australia noted that the “staff with the highest fidelity score is a senior Indigenous researcher who facilitated the establishment of trusting relationships and employed colloquial language, which strengthened relationality” [23]. This speaks to the need for interventions to practice local communicative practices and values in a trustworthy manner. Contrary to previous outcome, researchers in another study found that the practitioner with the highest MI proficiency throughout the study had the worst participant oral health outcomes [26].

With inconsistent tools to identify “good participants” for MI in OH education [28], difficulty producing practitioners with proficient MI skills [23], and inconsistent measures for understanding specific aspects of MI and how they impact ECC [26,27,28], we are left to continue testing MI in Indigenous communities—or tailoring MI to fit the community and desired outcomes. It should be noted, for the interest of this manuscript, that research [23] specifically identifies an Indigenous researcher who was able to use colloquial language in the intervention was able to strengthen relationships with participants.

### 2.2. Language Use, Communicative Practices, and MI in Public Health Interventions

Having studied MI in various environments for the last two decades, anthropologist Summerson Carr raises concerns about the practice of MI [29]. Critically, Carr notes the power inequity built into the MI empire. A pattern within the MI-practicing population involves those with elevated degrees recruiting and training others with elevated degrees to become authenticated MI practitioners (MINT members). These professionals are then paid through grants and professional funds to train helping professionals and researchers. These researchers and helping professionals often implement MI in marginalized and culturally diverse populations [35].

Carr proposes that perhaps the point of MI is to minimize cultural differences before the MI session begins. To clarify, Carr states, “dismissing marked difference from the start, as in claims that MI is globally applicable or when white male client-actors are left unmarked, standing as universal subjects” (2023, p. 23). By dismissing the possibility that some communities function differently, we erase the potential for meaningful difference to inform the motivational interviewing process itself. Carr further presses this blindness to difference by insisting that “the failure to adeptly deal with sociological differences—that is, inequities—and the suffering they cause should press upon us all here and now” (2023, p. 199). While MI is an American therapeutic method, the current practices need not to define (nor sell) their approach as a panacea. Carr summarizes this point illustrating MI as an American creation loaded with American ideologies by claiming, “MI is American to the extent that it fails to historically situate itself and its ways of seeing and speaking to others” (2023, p. 199). This ignores the differences within and between communities and professes a homogenized approach that will satisfy all limits the ways in which we are able to meaningfully communicate with participants in otherwise tailored interventions.

For linguistic anthropologists, how people speak and are spoken to is distinguished by cultural practices and beliefs embedded in language ideologies [12]. In his book, Native American Language Ideologies, Kroskrity frames language ideologies as “beliefs and feelings about language and discourse that are possessed by speakers and their speech communities” (2009, p. 4). Using this definition, a community’s ideas about language reflect that community’s understanding of language as a code (written) or language use as the norms and expectations in social contexts where there is speaking (talk). These beliefs and feelings can vary widely within and across NA communities [12]. This reiterates why it is important to allow for differences in communication and meaning making in context; these experiences are varied across the globe. Wollard reminds us, “There is as much variation in ideas about language and about how communication works as a social process as there is in the very form of language” [36]. The practice of MI is grounded in an ideology that (unintentionally) ignores these differences, privileging one system of communication while marginalizing another.

Local language ideologies influence language socialization, interpersonal communication, identity formation, storytelling, and other ways in which language is used and understood. Nevins’ work with a White Mountain Apache language revitalization program illustrates how these ideologies can shape the outcomes of language learning initiatives [37,38]. In this case, elders opposed the use of technology-based tools to teach the Tribal language, believing that such methods contradicted traditional ways of learning. The resulting conflict between elders’ and youths’ language ideologies ultimately led to the program’s collapse. For the elders, language learning was inseparable from interpersonal interaction and practice; they viewed the tech-based approach as incompatible with these values. In her book, Nevins discusses how the community talked about these issues: “Apache” versus “White” ways of speaking, behaving, and acting [15,39]. Though the program failed, knowledge about the importance of language ideologies and its role in language learning surfaced [37].

Closer to the intersection of anthropological understandings of communication and public health messaging, Charles Briggs reminds us of all the ways language and health communication can be used as tools to help or harm in medicine [40]. Briggs reminds us that language serves as a complex interface between languages or communicative practices and becomes a reconstructed representation of speakers. This can be seen in the way Americans routinely assign inferior status to Spanish speakers [41] or in the ways racial profiling has become medical [40]. For Briggs, he has noted how what he calls “health/communicative inequities” not only shape how care happens, but also how it is discussed and written about. When healthcare or public health interventions fail to recognize or incorporate local communicative practices and language ideologies, they unintentionally marginalize other forms of knowledge and care. Applying Briggs’ framework suggests that without *communicative justice*—or equitable recognition of how partnered communities construct meaning, express care, and communicate themselves—health communication and interventions, such as MI, may fail to resonate with certain populations. This disconnect can hinder trust, obscure health goals, and reproduce inequities under the guise of evidence-based practice.

## 3. Setting

GBHNS partnered with two rural, Tribal communities. One community is a Northern Plains Tribe and the other a Southwest Tribe. The two Tribes share some characteristics: they are relatively smaller Tribes (less than 15,000 members) located in rural regions of the United States, with a high prevalence of ECC and limited OH resources. The Northern Plains Tribe has an Indian Health Services (IHS) hospital and clinic, a Bureau of Indian Affairs (BIA), and the Tribal government and programs. While all enrolled Tribal members are eligible to seek dental and medical services at the IHS facilities on the Tribal nation, those who live off the Tribal nation often must travel a long distance (at least one hour away) for medical and social services. During the time of the intervention, the Northern Plains community had a federally funded Nutrition Program for Women, Infants, and Children (WIC) that helped families with healthy food. Their Tribal Head Start Program had lost funding and was not functioning.

The Southwest Tribe is also very rural and people who live on the reservation often need to travel 30–40 min for OH care at the IHS clinic on the reservation. If in need of specialty care, they may have to travel as far as 150 miles. The Tribe’s WIC program also assisted families by providing access to healthy food. Unlike the Plains Tribe, the Head Start Program was functioning during the time of the intervention, assisting 200 primarily low-income children ages 3–5 with access to education and services.

## 4. Methods

Over the history of the project, questions and concerns about the appropriateness of MI were raised by intervention staff. Adaptations to MI were made, allowing the concerns to be addressed by the research team and MI consultant (explored in 6.3). Having been part of the GBHNS research team from 2019 to 2025, our notes and observations from years of training and meetings came into play in the interview development. This allowed for interview guides to be informed by participant observation. We chose to use semi-structured interviews for data collection for several reasons: (1) they allowed for more discussion-based interviewing techniques; (2) they allowed each participant to be asked a similar set of questions combined with the flexibility to adjust to the natural flow of each interview based on what was determined significant by the participants; (3) they allowed the researcher to incorporate the participants own words; and (4) they allowed for generation of comparative data. With this approach, we were able to return to issues raised throughout the course of the project. It was important to allow for differences in the interview structure as each MI staff member experienced MI differently in the field, overall, and with other projects.

Interviews with 3 CHRs, who delivered the intervention, and 3 MI Leads for this project were conducted post-intervention. The 6 interviews accounted for 100% of the MI staff in 2024 when the study ended. CHRs and MI Leads were invited via email to participate. Interviews were conducted on zoom. All CHRs and MI Leads from GBHNS agreed to participate. All but one interviewee identified as Indigenous. Consent for the recorded interviews was collected via REDCap [42]. All interviews were recorded and then transcribed with Trint.com and reviewed by an NAU researcher for accuracy. All transcribed interviews were uploaded to a secure server and coded with ATLAS.ti [43]. Interviews were coded thematically by a qualitative researcher using an inductive and deductive approach [44]. Themes were generated based on interview questions (i.e., What would you change about MI? What do you think is the value of MI?) and data-driven themes of experience (i.e., metalanguage, problems with MI, dealing with MI issues, discomfort, communicative practices, adaptations). All data presented is directly from the transcript, without summarization or rearranging interviewees’ words. A limitation of the qualitative design is the post-study interview.

### Positionality Statement

Having worked with the GBHNS team for the length of the project, the lead author occupied a unique space as a researcher trained in linguistic anthropology and working in public health. Through observations of the years-long MI training, her background in linguistic anthropology became a unique lens for considering the metalanguage expressed by the CHRs. Previously investigating the co-constructed pregnancy experience of first-time parents, doctor/patient clinical conversations, and now MI, she took special note of the metalinguistic comments and concerns reported by CHRs. She found herself in a space between two disciplines—experiencing the tension of public health intervention protocol and her anthropological thinking and training. Over time, the CHR concerns grew—leaving her to struggle with bringing together two disciplines. Returning to research and theory on language use and language ideologies to investigate the concerns raised during the project, she then was able to identify a disconnect (or referred to here as a ‘border’) between the communicative practices of each community and the claims of motivational interviewing.

## 5. IRB

The Northern Arizona University Institutional Review Board approved this study on 21 December 2022 (IRB #1920796-6). Informed consent was obtained for all interview participants using REDCap [42].

## 6. Results

As team members of GBHNS and having worked alongside the CHRs and MI Leads for the length of the project, all authors had good relationships with the staff at the time of the MI expert interviews. The interview guide asked specific questions about motivational interviewing and staff experiences (in the case of GBHNS and any MI research experience outside GBHNS), it became clear that each interviewee had several recommendations for how to make MI more appropriate for their communities. The lessons learned included five main themes: (1) the importance of trust in communication; (2) MI communicative issues; (3) adaptation of the GBHNS MI intervention; (4) positive impact of MI; and (5) MI proficiency and intervention requirements: staff-recommended adaptations and metrics.

### 6.1. The Importance of Trust in Communication

“Trust” was discussed in all six expert MI interviews. Trust appeared especially important in cross-cultural MI interventions and within Indigenous interventions. As a practitioner with years of MI experience, a MINT member, and MI educator, MI Lead 3 shared just how important trust building is for any cross-cultural MI session:

*I had Native students bring up differences in culture, especially when it came to the engaging process, and they said, you know, in our in our Tribe, especially if somebody is counseling across cultures–so a white person counseling and meeting in person--there’s so much distrust from historical trauma that the amount of time that’s needed to build engagement is exponential. And it may be an entire session where you have to just stay in the engaging process or multiple sessions to build that trust first. And also at the same time, other groups are in a big hurry to get things done and are very task oriented. So like white people are sort of notorious for like, “Let’s go. Let’s get the education now. Let’s do the thing. Let’s move on. Check.” And that culturally, that’s not an Indigenous way. And so there’s that piece too, of like there’s relationship building, there’s no hurrying, and so that was maybe the first time that I had a conversation with someone who was not white about the contextualization of my two other groups, and I just value that so much.*

[MI Lead 3]

Crucially, MI Lead 3 went into detail about the trust-building strategies depending on the populations involved. It should be of note that this cross-cultural information provided to MI Lead 3 was not the result of MINT MI training but from Indigenous students enrolled in their course. Stated plainly: A certified MINT member did not learn about cross-cultural trust-building practices in required MI testing. While the 2023 edition [32] has begun to acknowledge cultural adaptations of MI, the experiences of GBHNS’ participants suggest that there is still significant work to be done in translating these principles to specific community contexts. This reinforces concerns about motivational interviewing’s supposed universality and lends support to Carr’s (2023) [29] critique that MI lacks the capacity to meaningfully situate itself within diverse historical and cultural contexts. Ignoring cultural and communicative practices which build trust with clients dismisses necessary steps in rapport building and could crucially impact how participants engage with public health interventions.

As MI Lead 3 describes above, participants may need time to generate trust with their MI practitioner or CHR. In a similar recommendation, MI Lead 2 echoes the importance of trust building and even describes experiences from GBHNS CHRs to illustrate the difference trust can make in an MI oral health education session:

*I would basically say that trust is really important within MI and that’s something that’s not really in the textbooks at all. Like that’s all kind of like thrown under the realm of like rapport building I think is like developing that trust. But… I think hearing that the CHRs have basically said that they had the better sessions with people that they kind of knew already. That just kind of tells me that the participant went into it kind of already trusting what was going to happen. And I think that makes a lot of sense and kind of like what I was hearing because like, there is just like a lot of like hesitancy kind of like behind the mothers that they didn’t know… Yeah, it’s like a trust thing, you know, because like a lot of the conversations that do come up in MI sessions can be very personal at times.*

While MI literature addresses trust under the umbrella of “rapport building [18],” the depth of culturally specific trust building practices described by GBHNS participants suggests that this treatment may be insufficient for cross-cultural contexts. It is important to know how rapport is generated within communities and through what specific forms of communication. After leading bi-weekly MI trainings for CHRs in GBHNS, MI Lead 2 summarizes that CHRs who already had relationships with participants tended to have more trusting and better sessions; trust had already been built and they did not need extra time and space to generate it. Although often overlooked in the planning stages of public health interventions, designing interventions that allow adequate time and space for trust-building between CHRs and participants could lessen the burden placed on research staff to establish rapport within the constraints of a fast-paced intervention timeline.

### 6.2. Examples of MI Communicative Issues and Concerns from the GBHNS Project

While training CHRs for study launch, GBHNS scheduled multiple group MI trainings. Bi-weekly MI trainings occurred from 2022 to 2024 with weekly homework and MITI assessments. An in-person All-Hands meeting also occurred at Northern Arizona University in 2023 prior to study launch. During this in-person training, several concerns were raised about aspects of the practice of MI. One CHR was very vocal about the ways in which they would and would not communicate with members of their community. Communication about communicative practices (metacommunication) is incredibly pertinent in gleaning accurate information from participants [16]. When asked to reflect on their specific comments and concerns from the All-Hands training, CHR 1 responded:

*So… we did the training …and maybe it was [their] approach that made me not feel as comfortable with it and watching the videos that [they] did. It came across to me, my initial reaction and… It sounds so forced and, um just to be blunt, it sounded fakey to me and I didn’t like it. I didn’t like it. I thought, this is not a natural way for me to talk to my community. It sounds um unnatural. That was my reaction to it. In all honesty.*

[CHR 1]

Concerns of communicating with sincerity and authenticity were frequent comments during MI trainings. CHRs were pointing out the difficulty of educating their community while feeling they were being “fake” or talking in a way that would be considered inappropriate. CHR 1 describes their uncertainty in speaking to people in a way that did not seem natural for interacting with others in their community. This discomfort with stepping outside the norms and practices of their speech community for CHR1 highlights their beliefs about how talking naturally occurs in their community.

Given that MI involves open-ended, emotionally engaged communication, it can at times become emotionally taxing for those implementing it. During the intervention period, several news outlets reported the discovery of mass Indigenous burial sites. Participant reactions to these events often emerged at the beginning of sessions, particularly when CHRs initiated rapport building by asking participants about their day. Sometimes this general open-ended question caused CHRs to not only initiate the public health intervention, but to also listen and care for a distressed community member. One CHR described their experience:

*So if it’s like, that [burial sites] came up… if you want to discuss that… I’ll be willing to listen. And that’s how I handled it. I concentrated on what I was there for first and then… I sat and I listened. I didn’t, you know, I didn’t try to give advice. I only recommended resources that they can reach. Some of them were more severe than others. But I tried.*

[CHR 3]

As an unanticipated emotional topic outside the realm of oral health, this CHR reported doing their best to listen to their community members when these concerns came up.

When asked if additional practice with a focus on dealing with distraught participants would have helped during the intervention, CHR 2 responded:

*<exhale> I think it should be brought up and I think it should be talked about so that it is there and it’s not something that freaks them [practitioners] out (laughter). You know, it’s traumatic for someone who first is doing something that is feeling overwhelming, and they really don’t know what to do with themselves. You know, that’s really traumatic and stressful… So if we talk about it in the beginning, we talk about it and, you know, learning about it and how to deal with it a little bit, I think the individual [practitioner]… will gain the knowledge and tools to utilize that as well as the MI.*

Further, an MI Lead with experience in other Tribal community projects echoed:

*Like it’s hard to not emotionally invest yourself into what [participants are] going through. And I think as Native people and Tribal communities, I think, we have that tendency to do something like that. We want to listen and try to help in a way that we can without trying to get emotionally invested. But that’s never the case. You somehow, get, um, attached to it. And in this case [historical trauma], in MI, I think people have that opportunity to unpack if they if they want to. But then it’s also the MI professional who then kind of takes a piece of that with them… And I think I think in our case with MI and Tribal communities, I think that’s something to consider.*

[MI Lead 1]

Both CHR2 and MI Lead 1 called attention to the specific unanticipated emotional work of using MI in a public health intervention. While CHR 2 expressed the overwhelming experience of being on the receiving end of unanticipated traumatic conversations, MI Lead 1 added longer-term emotional concerns. Working within your own community while using MI means putting yourself in that person’s shoes and carrying their experience home with you.

### 6.3. GBHNS MI Intervention Adaptations

As stated, the practice of MI is based in patient-centered therapy. Therapy sessions tend to have space for following a participant’s desire to discuss whatever they may need for the purpose of their therapeutic health journey. When using MI to educate participants on a specific health topic, the space for topics relevant to the participant becomes more narrow. For example, if conducting an oral health intervention, one would want to focus on fluoride use instead of triggers for what causes one to binge drink (a clinical use of MI). For GBHNS, several practices within MI were altered to accommodate the culturally tailored oral health intervention session topics. For example, GBHNS used scripts designed to help CHRs work their way through session topics selected by the participant. The use of scripts goes against the practice of MI in that it becomes less of a free-flowing session and more structured. However, criticism of MI in public health interventions has noted the inconsistency in benchmarks for educational topics covered during MI sessions without scripts [23]. Throughout GBHNS session development process, scripts were edited by Community Advisory Boards, CHRs, oral health providers, and MI staff. One edit to scripts stemmed from the issue previously covered by CHR 2 (i.e., unanticipated historical trauma from open-ended questions in the rapport building stage). To narrow the scope of the discussion to oral health, the GBHNS team altered the scripts so that open-ended rapport building prompts focused more on family oral health practices.

As a result of the shift in study objectives necessitated by the COVID-19 pandemic, the GBHNS intervention was modified to include only a single oral health session with each participant, rather than the originally planned six sessions over a three-year period. This significant reduction in contact, may have further limited CHRs’ ability to build rapport with participants over time. With only one session for building rapport, CHRs and MI Leads made the decision to eliminate the tracking of “complex reflections” as these types of reflections involve in-depth (and long term) knowledge of participants, their lives, their struggles, and their goals. Complex reflections are a behavior count item on the MITI scoring system and defined as reflections which substantially add meaning or information to what the participant said [34].

While the removal of complex reflections from the scoring criteria was a study-specific adaptation related to the intervention format, additional modifications to the MITI were made with consideration of the linguistic and communicative norms of the participating speech communities. Specifically, GBHNS did not count “affirmations.” For example, reflecting on experiences in another Tribal community using MI as an intervention, MI Lead 3 discussed how “affirmations” were received by participants:

*… we did a MI training with [Tribe]… And then we learned a few more pieces from them. One being that affirmations were not something that that happened a lot in just naturally in their culture. And so they definitely weren’t open to delivering affirmations.*

[MI Lead 3]

GBHNS CHRs also reported that, during their homework assignments (short MI sessions with friends or family members), affirmations were confusing to their participants and often caused them to shut down. MI describes affirmations as, “a clinician utterance that accentuates something positive about the client. To be considered an affirmation, the utterance must be about client’s strengths, efforts, intentions, or worth” [34]. GBHNS advised CHRs to “tread delicately” when discussing participant strengths or efforts and, if in doubt, to avoid the practice altogether and instead reflect upon participant stories.

### 6.4. Positive Impact of MI

Each MI Lead working with GBHNS had also worked with other projects or groups implementing MI. A question posed only to MI Leads, inquiring about “the value of MI,” resulted in responses that spanned more than the years of training with GBHNS. As Indigenous MI Leads, or Leads who had worked with other Indigenous projects, querying the value of MI may help researchers in public health understand the possibilities and limitations when using MI in Indigenous communities.

Overall, MI Leads did not dismiss or discount the practice of MI. In fact, they passionately discussed how helpful MI can be when working within Indigenous populations. Leads discussed the understanding that can be achieved with MI, along with the ability to ‘just talk through issues’ with someone. One Lead reported:

*I think the value of MI is just, knowing—who—you are having this conversation with and kind of going on that journey together with them and helping them—helping them unpack, um, so to speak. Kind of how they want to make a change in their own lives. And kind of talking through those different steps. I think having someone there and kind of—is the word sympathetic or empathetic with the person? I think that’s what kind of really, might be the main value of MI in my opinion.*

[MI Lead 1]

For MI Lead 1, central to effective motivational interviewing is the act of building a social relationship through performing empathetic communication—focusing intentionally on the person one is engaging with and collaboratively working alongside them. Generating empathy and sympathy is a process which takes time, space, and trust. Focusing on how MI can help people “unpack” and “make a change in their own lives,” this Lead returned to the central ideology behind MI, “helping people talk themselves into change” [18]. MI Lead 1 really dived into the structured proficiency criteria of the MITI aspect of MI, working with a participant through their day-to-day practices, what those practices meant to that individual, and how to move forward together with making healthy changes to those practices.

Another MI Lead, when asked the same question about the value of MI, focused on MI as an improved way to communicate with someone. For MI Lead 3:

*…the value of MI is it’s a way to love people better. Oh. And I know that’s so cheesy, but that really is what matters most to me is just how can we love people better. And love is a verb for me. Like how can we love people better in the way we talk to them? And I feel like, MI is the answer?*

Not focused on the outcome of behavior change, MI Lead 3 found MI to be an improved way of communicating with one another; a caring way to connect. Language as care is a culturally meaningful phenomena of social engagement. Focusing on how we talk to one another, for MI Lead 3, was the value embedded in MI.

Finally, with specific reference to GBHNS and public health interventions, MI Lead 2 highlighted the value of motivational interviewing as a method for gathering participant information through a non-medical, non-judgmental form of connection and communication:

*I think it just provides a little bit more of a connection compared to other ways of like acquiring this information… So, like instead of going to well going to the dentist since we’re talking about oral health and what we’re using it on, instead of like going to… a dentist and then sometimes, you know, you only see the dentist for like a minute or two. So, like, questions are asked or whatever. And then even just from personal experience, you know, growing up, if there’s ever like a major issue going on, it’s like ‘who kind of let this happen…?’ And that’s kind of like going back to that judgment thing. So, I think the benefit that MI really has is like it… provides like a space for like a connection… a true exchange of information…*

MI Lead 2 illustrated how interventions like GBHNS could benefit Indigenous participants by opening up an avenue for non-judgmental health communication. Here, Lead 2 indexed the judgment they perceive to be embedded in medical communication and highlighted that this judgment is not present in the practice of MI. MI Lead 2 focused on how MI communication creates space for connections and works with participants to gather information without judgment and blame, as they reported to have experienced.

In sum, GBHNS’ 3 MI Leads focused on how communication in the spirit of MI can reach participants in an empathetic, caring, and non-judgmental way. Each Lead commented on how MI as a communicative practice generates understanding and care. MI Lead 1 focused on how MI can push all of participants to care for one another better through conversation. Additionally, MI Lead 2 illustrated the ways in which communication with patients in the dental setting provides the opposite social interaction that MI offers. Lead 2 described their experience with dental providers as blame finders, using communication not to generate understanding or provide information, but to assign blame. The Leads emphasized that language use—and the relationships it fosters—varies significantly depending on communicative approach. They contrasted practices that facilitate information-sharing on equal footing with those that risk assigning blame or reinforcing hierarchies.

### 6.5. MI Proficiency and Intervention Requirements: Staff Recommended Adaptations to MI and Metrics

Engaging all six MI staff in a discussion on how motivational interviewing might be adapted for Indigenous communities, such as those participating in the GBHNS project, yielded valuable insights and actionable recommendations for shaping future culturally responsive public health interventions. Of note to CHRs and Leads, they discussed the following barriers to MI use without adaptation: (1) CHRs emphasized that MI is a highly specific practice that requires significant effort to learn and achieve proficiency and (2) they also noted that MI differs significantly from the ways they typically communicate within their communities. Drawing on their work with Tribal communities and MI, GBHNS CHRs and MI Leads offered several recommendations for adapting MI. While MI is often presented as culturally universal, staff experiences with GBHNS and similar studies suggest otherwise.

As an evidence-based practice (EBP), MI has specific fidelity monitoring protocols and procedures to be considered properly utilized or proficient in an intervention. To achieve proficiency, MI practitioners need to achieve a specific ratio of questions to reflections and be globally rated in a specific range for Cultivating Change Talk, Softening Sustain Talk, Partnership, and Empathy (a scale from 1 to 5) on the MI-validated measure, the MITI. Achieving a proficient score ensures that a participant experienced a true motivational interviewing session [45].

As CHRs in an intervention, not MI specialists, CHRs reported that they felt overwhelmed about all the requirements involved in MI to achieve proficiency. They expressed that not only did they have to learn to communicate in a way that felt “fake” compared to their usual conversational style, but they also had to meet specific MI requirements while simultaneously delivering oral health information. When asked whether MI could be effective in other Tribal populations, one CHR highlighted the overwhelming number of tasks required during an MI session in order to meet proficiency standards:

*It’s just like, “It’s got to be just this way. It’s got to be just this way.” You’ve got all you’ve got to do. I mean, all of that. You kind of sense when you are overdoing something. You know what I mean?*

When asked more about why they felt so pressured by the requirements of MI, CHR 1 continued:

*But I understand that that’s what we put in the grant and that’s what we have to do. I understand that. I’m not trying to mess up the grant …And if we come up with, you know, an indigenous approach to MI, I probably would be okay with that. But not the way it is right now. And I think that in any kind of research or interviews. You would want to do that, whether it was MI or whatever, you would want to do it in a way that would be most um successful in the community.*

[CHR 1]

CHR 1 recommended a different version of MI that might be appropriate for Tribal use and did not find it to be satisfactory, as it currently stood without adaptation. Referring to communicative practices in MI, CHR 1 found it inappropriate for their community; they recommended that MI should be utilized to be “most successful in the community.” This would require formative assessments to ensure that the values and communicative practices within MI resonated with the target community prior to intervention launch. Another CHR commented on the repetition of MI in order to meet study needs:

*You know, repetitive practice of using MI is very important in order… to be successful… And we did it. I don’t know. Even when I had all that, I did the extra training because I did other trainings outside of what was given through Healthy Native Smiles and um it-It had to be a constant repetitive use in order for me to feel comfortable of making sure I was asking or constructing the question to be open ended and to flow and to, um you know, gain the responses back.*

[CHR 2]

While CHRs understood the importance of implementing MI at a specific level due to the intervention design, they also appeared to question the appropriateness of MI for their communities and the demanding effort required to reach proficiency.

To ensure a better fit for each community, conducting systematic formative research on local communication practices would be beneficial for any public health intervention. Additionally, if the intervention involves an evidence-based practice (EBP) like motivational interviewing, understanding whether the values and approaches of that EBP align with those of the community could help prevent potential missteps. For example, MI Lead 1 recommends:

*I think if the project maybe did spend at least two months trying to discuss MI and even dig deeper into like, ‘how can we rephrase these questions or different MI concepts so that it works better for you or even for your community?’*

MI Leads also noted the need for formative assessments that not only identify the barriers and supports to oral health care in each community but also examine how the values and communicative practices of MI impact community members, would have strengthened the GBHNS intervention.

In a separate project during MI training, MI Lead 1 reflected on concerns raised by a different Tribal community—one not affiliated with GBHNS:

*It was like, well… “we don’t talk like this” or “we like to joke. We can’t be serious.” “We know a lot of these people who come and see us or talk to us,” so, um, I don’t- I think the fundamentals may have gotten across to that group… And I would confidently say that I don’t think it went well.*

[MI Lead 1]

Further concerns raised by MI trainees in a separate project, as described by MI Lead 1, suggested that participants perceived MI as distancing them from their own culturally grounded communicative practices—requiring them to engage in ways that felt inauthentic or inconsistent with how they typically interact with others. When faced with sacrificing joking communication, participants quickly outlined what practices ARE a part of their community (“we can’t be serious, *we like jokes*”).

Finally, MI Lead 1 deepened concerns for community research by indexing the impact of structural issues on Tribal communities. Addressing issues in educational materials, MI Lead 1 stated some families cannot meet the basics recommended for good oral health (i.e., transportation to care, supplies, fresh foods and water), so how can culturally adapted interventions better assist participants with these concerns?

*I think this study would have been better to kind of hone in on the specific community. But I also think maybe it should have started a little bit smaller with more realistic goals, with like the whole, flossing, brushing and even diving deeper to what are the actual environmental factors that may prevent someone from getting access to foods… Because I know that was one of the things that one of the sessions that was talked about in this project specifically was like sugary drinks and kind of drinking more water… There were a couple mothers who were just like, “That’s what I have. That’s what I can afford.” And it’s like, okay, we realize that there’s this other factor that’s playing into why they can’t afford or maybe even do X, Y, and Z. Um so I’m just thinking of like- and this is general for like all Tribal communities or any communities I think in this case it’s like there’s other factors that we need to consider um, because it’s not going to be like an easy fix, so to speak.*

[MI Lead 1]

Given the broader social determinants of health affecting each community, it is essential to tailor educational materials and motivational interviewing strategies to better equip CHRs in responding effectively when these structural limitations emerge. Griffith et al. discuss this as the “cultural sensitivity” approach for culturally tailoring health communication messaging [10].

When asked if there was anything else to add about using MI in their community, CHR 2 replied, “*People doing MI going forward, I think they need that multifaceted view, especially if they’re going to work in communities such as mine*.” For CHR 2, this multifaceted view includes considering adaptations to interventions at multiple levels (content, communication, practices) in order to fit them to communities—not only in regards to the health concern, but the way in which the concern is appropriately acknowledged and discussed.

Culturally tailoring interventions in public health is a beneficial step forward in making interventions impactful for specific populations. Acknowledging the shift in public health to better communicate health messaging with populations through the practice of culturally tailoring education and interventions, MI Lead 2 discussed the obvious move to also alter the MITI for similar purposes:

*I think probably since, you know, [I’ve] been studying a lot of the …tailoring something big down … and using it for a specific like community type stuff. And that’s really where public health is kind of like moving overall is like figuring out how to be better at that process. And so I think, MI still be[ing] used within public health like that just has to like follow along with, you know, the tailoring of like not being so nitty gritty on like the ratios of like questions to reflections type stuff. And I know like the reflections is something that is probably one of the things that I would be most interested in, like if we avoided reflections. Working with Native communities, you know, like that itself, I think probably would have made it feel a little less like a therapy session, I think that’s definitely one of the main things that kind of like catches people off guard, you know, when they haven’t experienced it and they’re being interviewed. It’s like this person’s like saying all my stuff back to me all the time, you know?*

The rigidity of MI, or at least the fidelity monitoring (constant scoring and reflection), puts pressure on staff across projects utilizing MI. MI Lead 2, in agreement with other Indigenous staff working with this project, expressed the possibility of moving forward with culturally tailored, even community-tailored, MI.

All staff agreed that alterations to MI and the MITI would better serve the communities discussed in these interviews. Regarding the need to locally and culturally tailor MI and assessments, MI Lead 3 discussed how these might be adapted:

*Yeah, I do think there should be more wiggle room in the MITI coding… The whole question to reflection ratio is a great example of why you can still sort of be out of alignment with a 2 to 1 ratio and actually deliver a session that’s fully in line with the spirit of MI. But that’s maybe one reason why MITI coding--there isn’t a single MITI coding score, right? There’s just these different scores and those global scores represent the spirit of MI. And so I think with even within projects, you could say, you know what, we’re only going to look at the global ratings score to determine, MI fidelity in this project? And maybe that would provide some more open translation in a cultural context of MI? So that’s a case for what you’re saying on the other side of that. I do feel like in a lot of cultures, reflective listening demonstrates empathy, which is a part of acceptance. But if that that doesn’t translate to your culture, then yes, maybe that needs to be tweaked. But yeah, it’s really challenging because the person who… identifies from a different culture, from the trainer could easily just say this reflective listening stuff is nonsense. Like ‘this would never work in my culture.’ But is that true or is it just so different from how they’ve communicated before that they’re making an assumption that it wouldn’t demonstrate empathy?*

As a MINT practitioner, this trainer’s response not only reflects their experience with GBHNS but across other programs and projects with Tribal communities. At the end of the excerpt above, MI Lead 3 called into question specific communicative practices (reflective listening), how those practices are performed and received in different cultures, and how to go about uncovering that answer. To better understand how the core values of MI are received within partnered communities, research teams should collaborate closely with those communities to assess how MI principles and practices are interpreted, enacted, and negotiated across cultural and linguistic contexts. While reflective listening, to this lead, does demonstrate empathy—practitioners in GBHNS have commented during team meetings that some participants respond to reflections as if practitioners are not paying attention to their perspectives.

While these recommendations from MI staff focus on adaptations to the MITI, which is essential in fidelity monitoring of clinical trials and public health interventions; the alternative is significant formative research in communicative practices of local populations prior to introducing any “talk based intervention.” Learning how specific communities communicate values like listening, mutual respect, understanding, and how to share information is essential in the creation of any attempt to monitor communicative success. Returning to researchers who have also recommended changes to MI for Indigenous practice [19,20,33], we echo the need for MI to address inherent power imbalances [29], acknowledge the limitations of Western methods in capturing Indigenous knowledge and perspectives, ensure mutually supportive partnerships, recognize and uphold Indigenous ways of knowing, being, doing, prioritize Indigenous self-determination and prioritize Indigenous-led approaches. We also support the immediate need of capturing contextual relevance and nuance [19]; in this case, the team would like to especially highlight the contextual relevance and nuance of speech communities and communicative competence.

## 7. Discussion

The data presented in this manuscript are intended to illustrate the lack of permeation of “cultural tailoring” in public health interventions to all areas of health, culture, and practice. The evidence of the borders of “cultural tailoring” appeared in the concerns from CHRs, highlighting communicative misalignments. As Briggs (2017) [40] labels this type of phenomenon “health/communicative inequities”—in this manuscript we point to an example in the culturally tailored public health intervention utilizing motivational interviewing. While education materials within interventions may be tailored to reflect local values and beliefs, other aspects of interventions (like spoken language and dyadic communication in interventions) can be left to carry the values and beliefs of the Western medical model, American ideologies, and other hegemonic practices.

GBHNS staff illustrated the importance of generating trust within their communities, communicative issues they experienced during this research, adaptations they would recommend in future research like GBHNS, and their experiences of the positive impact of MI. In addition, we presented data to illustrate the specific adaptations GBHNS made to MI in order to better attend to CHRs and the participants along with recommendations regarding the cultural tailoring of MI and downstream research issues. In this discussion, we will further explore these topics and recommendations for introducing more salient communicative practices within public health interventions.

As discussed, MI does not specifically mention “trust” as a central value in its practice, but it was clear to MI staff of GBHNS that trust was a central value to participants and CHRs. For CHRs, trust came in the form of bringing OH education to participants’ homes and communicating that education in an authentic and appropriate manner. Expressed throughout training, the intervention itself, and the in-depth interviews presented here, MI as it stands did not satisfy all CHR needs for building trust with members of their communities.

Building trust through conversations can be as simple as communicative salience. Talking with someone who reflects similar values and communication styles can be affirming. Silverstein defines linguistic ideologies as “sets of beliefs about language articulated by the users as rationalization or justification of perceived language structure and use” (1979, p. 193). When the CHRs were expressing to the GBHNS team during training, “*We* would never say *that*!” (author’s emphasis), the metacommunication here clearly outlines what is considered appropriate for this community and what is not. If public health interventions continue to use the rigid MI structure instead of incorporating more locally relevant linguistic practices which reflect local values, intervention practices could limit the normal flow of conversation, which would otherwise illuminate the experiences and concerns of target populations in Tribal communities [16].

Although the single-session GBHNS intervention resulted in statistically significant knowledge gains in both communities [46,47], future efforts should consider implementing multi-session (longitudinal) formats. Expanding the intervention would enhance CHRs’ confidence by providing more opportunities for in-depth participant engagement, rapport building, health education, and the development of personalized change plans. Returning to how trust is generated in the partnered communities, CHR 2′s comments also focused on the time it takes to build trust with their community members. The ability to generate trusting relationships could positively impact session outcomes by increasing knowledge and preventative behaviors of participants.

MI Lead 1 drew attention to the practice of care within Tribal communities (“*going on that journey together with them and helping them*”). Researchers have demonstrated that in Indigenous communities, the community itself is part of health [4,5]. For example, Wexler and Gone have called for culturally appropriate public health interventions regarding suicide and self-harm. They observe that Western psychological frameworks, which emphasize individualism, are embedded in many treatment models—an emphasis that often conflicts with Indigenous conceptions of health, which are collective and relational. Aspects of MI (like Emphasizing Autonomy) may violate local understandings of collective health and wellbeing. Formative assessment data also illustrated collective health in the form of the importance of the family and perhaps even extended family in oral health practices [3]. Preliminary research in communities to identify concepts of care, health, and wellbeing, and how best to communicate these concepts through educational interventions, would benefit communities and participants.

Additionally, MI Lead 1 discussed the need for more in-depth formative work on how deep SDOH impacted these communities (not having access to fresh foods, water, or basic OH supplies). For GBHNS, this could look like additional training or prompts within session scripts to help brainstorm with caregivers in these delicate states. A lack of consideration for this type of issue could also reflect the beliefs of public health researchers creating oral health interventions. For some participants, “basic” supplies like clean water, toothbrushes, and floss may not be considered basic to participants due to structural issues embedded in their communities. Formative research covering appropriate ways to discuss oral health from an acceptable entry point would allow participants to feel safe, not judged, and capable of working with CHRs to improve their at-home oral health practices with what is available to them and their family.

Ultimately, with more formative work focused on how to discuss oral health care while considering local norms surrounding oral health practices, CHRs may be able to implement a version of MI that more closely aligns with community-specific communicative practices. As a product of Western psychology, MI was originally developed as a therapeutic approach characterized by open-ended, client-centered dialog that creates space for individuals to explore meaningful topics at their own pace. However, when MI is adapted for use in public health interventions—such as those focused on oral health education—it often requires additional structure and programmed content delivery. This shift introduces a tension between MI’s original person-centered therapeutic intent and its implementation within more goal-oriented intervention structures. As GBHNS worked through creating sessions, iterative feedback from CHRs and CAB members was invaluable.

Local communicative practices and values are especially important in Indigenous communities, as their local knowledge has been neglected or pushed aside when struggling with the experience of external cultural influence [12]. As Carr (2023) [29] discussed, MI is an American therapeutic tool imbued with American ideologies and practices (like ignoring differences and historical relationships). One approach for adapting public health interventions includes a focus on decolonizing education and research. This practice has gained support in recent decades and both topics are important when focusing on language use in public health interventions within Indigenous, NA/AI/AN communities [5,48]. Decolonizing methodologies can also be described as Indigenous methodologies [49]. Implementing a decolonizing approach “brings into clear view ways in which power/privilege/oppression are reproduced and contested through racialized and ethicized practices and discourses” [50]. This can also be seen when language revitalization efforts by academics or top-down efforts fail as opposed to local grassroots movements that are more integrated into the communities in which they arise [37,51]. After centuries of assimilation, removal and relocation, it is understandable that Indigenous communities are deeply suspicious of efforts involving their people or their languages in academics.

### Using MI in Communities Moving Forward: How to Adapt

Reinforcing local Indigenous ideologies and communicative practices throughout public health interventions could help mitigate the feelings of distrust or inauthenticity [5]. In addition, implementing these practices and values could provide more sustainable outcomes for specific Tribes or populations as it could be adapted and applied across healthcare concerns. Calls for adapted interventions to better suit communities is not a new request [48,50,52,53]. GBHNS MI staff provided recommendations for creating a manageable workload for local CHRs to implement along with possibilities to better assess MI proficiency in communities.

Data revealed that CHRs reported problematic experiences with aspects of MI. Most notable in this manuscript, CHRs and MI Leads reported issues with complex reflections, affirmations, and a lack of incorporating local communicative practices (like joking). While MI Lead 3 and Leads did not systematically explore the communicative practices affirmations appeared to violate, further research in this community about why affirmations are uncomfortable could lead to alternative communicatively salient practices or merely altered guidelines on implementing MI in this community. Following metacommunicative feedback from MI Lead 1′s research population (‘we like jokes’) and finding ways to incorporate what *is* part of this community could lead to improved public health interventions using MI. All three MI Leads agreed that pulling MI out of the rigid clinical therapeutic realm and applying more locally congruent values may better satisfy participants in these communities [23].

CHRs reported feeling overwhelmed regarding the rigid practice of MI while also delivering oral health information. Though GNHNS designed adapted MI scripts for each of the six culturally tailored oral health sessions, perhaps future work could assist in making the scripts more helpful. The GBHNS’ scripts assisted CHRs with covering all sections of oral health education in each session. Prompting CHRs to practice MI values (i.e., reflections, open-ended questions, Seeking Collaboration, Cultivating Change Talk, Softening Sustain Talk) at poignant moments during the education session and during the goal setting conclusion of the session, scripts also assisted CHRs to reach MI goals. Though scripts are not “in the spirit of MI” [29], these scripts increased the consistency of oral health education for participants across sessions and addressed previous concerns with measuring the impact of MI on ECC [28].

Criticism of MI proficiency in Indigenous oral health education interventions alongside insufficient capabilities to test efficacy are frequent [23,26,54]. Proficiency is a helpful tool in the scientific process of replicability, but efficacy in MI interventions is plagued by inconsistent practitioners, a lack of standardized educational benchmarks, competing treatments (this would include education, MI, and practices like applying fluoride), and participant readiness to change [28]. By implementing scripts and benchmark-related oral health knowledge, interventions could generate more consistent practices for implementing adapted MI with oral health care interventions.

Taking advice from GBHNS MI Leads and CHRs, loosening the strict ratios and calculations involved in the MITI may better reflect the quality of MI in culturally tailored oral health intervention sessions. Starting with MI Lead 3′s recommendation of focusing on the MITI’s four global scores (Cultivating Change Talk, Softening Sustain Talk, Partnership, and Empathy) instead of the ratios could reduce CHR overwhelm. Allowing CHRs to focus on building good relationships within their communities instead of worrying about the behavior counts involved in MI proficiency scoring is critical; oral health education and participant wellbeing and the wellbeing of the broader community should take the forefront of sessions. However, issues would still be present within the MI global scoring system. Empathy is an interesting concept to “score” when working within your own community—if you already lead a similar life to your participant, how hard would you have to work to grasp or understand your “client’s” perspective? In addition, would cross-cultural scorers be able to identify all ways in which communities express partnership or empathy? Ultimately, the need to alter assessments to better understand MI within diverse communities illustrates its lack of preparedness for working within these communities.

Any cultural adaptation to the MITI would render the validated measure useless. The lack of a validated scoring system for adapted MI sustains the use of the MITI across populations, without cultural and communicative adaptation in public health interventions. Inconclusive results regarding MI and ECC reduction in Tribal communities is well documented and the inconsistencies could be attributed to the concerns and communicative issues discussed throughout this manuscript [23,26,28]. The absence of a generalizable measure of MI “proficiency” within speech communities whose communicative norms differ from those embedded in MI does not indicate a failure to generalize the practice itself. Rather, it may reflect a reliance on counting practitioner behaviors within one-sided interactions that do not necessarily capture the spirit of MI in a session.

While adapting the MITI may compromise the generalizability of MI across populations, it also presents an opportunity to develop new assessment tools in collaboration with linguistic anthropologists, medical anthropologists, sociolinguists, and local cultural experts. Such tools could better capture conversational practices across cultural contexts. Through systematic observation and in-depth communicative research along with iterative feedback from community members, new frameworks for evaluating MI may emerge. Incorporating the diverse values and communicative practices that distinguish communities not only enriches research but fosters more respectful, culturally aligned public health interventions. Supporting communities by using their own communicative practices to promote at-home oral health honors their values, practices, and cultures of individuals and families.

Finally, without special attention to differences, especially regarding social, structural, and individual practices, how do public health interventions expect to address unique population needs? As Carr [29] criticized MI’s standard practice of dismissing difference, dismissing differences also overlooks structural issues and cultural practices which may have impacts on important practices central to interventions like GBHNS. For example, discussions about structural and cultural practices surrounding access to oral health care would be exactly the discussions CHRs and participants need to engage for improving oral health practices. Additional recommended adaptations to MI could be made with adequate formative research prior to intervention launch. Ultimately, MI Lead 1, 2, and 3 recommended working with communities to test MI values and acceptability within the community prior to public health intervention design.

## 8. Conclusions

Considerable feedback from MI staff urged additional tailoring to the practice of MI even in the face of overall study success [47]. The GBHNS MI staff recommend considerable research within communities to explore practices and values of MI. This would truly begin to generate a people-centered approach to MI, ensuring intervention practices are shared by the community. CHRs reported considerable pressure from the rigidity of MI as a practice, discomfort with the “unnatural” communicative practices involved, and reports of MI practices that were not well received in communities (i.e., affirmations and complex reflections). Though proficiency is central to the practice of MI, GBHNS MI staff recommended several adaptations to the MITI for the sake of better assessing the quality of MI in Indigenous education sessions. These recommendations largely focused on utilizing the global scoring of MI over behavior counts and ratios. Additional improvements would include consideration of who conducts MITI scoring, as cross-cultural assessments of empathy and partnership (among other items) are problematic. Assessing whether CHRs were successfully able to cultivate MI’s definition of change talk, soften sustain talk, and encourage partnership and empathy, may be more in line with “the spirit of MI” [29] over concerns of proficiency in a public health intervention educational session. In the end, new forms of evaluation may be better suited for cultural adaptations of the practice of MI as any adaptations to the practice render the MITI inadequate for generalization.

In future interventions, disseminating community-approved scripts or intervention guides among projects like GBHNS would also benefit previously posed questions of consistency, efficacy, and relevancy of oral health educational benchmarks [23,28]. Minimizing aspects of MI which inhibit the building of trust or ignore cultural practices in specific health arenas could lead to a more salient practice of an adapted MI in Indigenous communities (or any specific speech community). It should be noted that this type of work should be done for each Indigenous population at hand, as no one community is identical to another.

For public health interventions working within Indigenous communities where dyadic communication is part of the intervention, it is important to understand the meaning of practices within the speech community. Using linguistic anthropological methodologies to uncover ideological beliefs prior to designing and launching an intervention in Indigenous communities would allow interventions to become more community-centered and culturally salient. MI as a vehicle for language is a social process that constructs identities and social relations and reproduces power relations. Therefore, when a participant experiences incongruent speech patterns, the establishment of trust between MI practitioner and client may be inhibited. Best practices should include substantial formative assessment grounded in interdisciplinary methods, with a focus on local communicative practices, knowledge, and values—particularly how trust and the principles of MI are enacted in conversation, including attention to underlying language ideologies.

Also important in anthropological research is reflexivity—not solely the anthropological meaning but the impact of research on others. When doing intervention work with communicative methods, it is especially important to understand the language ideologies of the researchers in addition to the ideologies of the community at hand [11,53]. Researchers can misconstrue meanings of findings and practices by asserting their own ideologies and communicative practices on others [55]. It is important to allow for space in understanding and sorting through these differences and allowing for the time this process may take (see MI Lead 3′s quote about cross-cultural trust building).

The uniqueness of linguistic anthropology and its methods to investigate the linguistic and sociocultural notions embedded in social systems (i.e., communicative practices, language ideologies, language use) should be considered a necessary framework for comprehensive interdisciplinary work with public health interventions like GBHNS [12]. Investigating local communicative practices *and* those of MI would be important for future studies. While MI has been implemented as an innocuous (sterile, scientific) EBP, it has its own ideologies and practices. Examining how language ideologies and communicative practices interact within and across speech communities is a distinct application of linguistic anthropology. As Wilce [15] describes:

Linguistic anthropologists are committed to finding out and giving priority to what social actors think they are doing together by looking at what they are doing. So we carry out our research not by asking these social actors what they are thinking, but instead by *analyzing what they do*—especially what they do *by saying (p. 146).*

MI, like any speech community, has its own beliefs about how language is socialized, practiced, learned, taught, even if it claims to be from the “same heart” as all humans [56]. The recommended linguistic analysis is in addition to oral health-focused formative assessment work such as that implemented by GBHNS (social determinants of health research prior to community involvement in culturally tailoring educational materials see Elwell et al. 2021) [3]. We specifically recommend linguistic anthropological methodologies which would include systematic data collection, discourse analysis, conversation analysis, and deductive and inductive qualitative coding and analysis [44,57,58,59]. Plentiful data collection through naturally occurring conversation about oral health practices, discussion of values and practices utilized in MI, and how those values and practices are performed and received, along with local perspectives on ideologies of oral health, language, interventions, would be necessary. Iterative methods following up on results or perceived practices would further clarify communicative practices, beliefs about oral health, and health values. Distinguishing and implementing communicative salience in public health interventions would benefit local populations and generate information on communicative practices for future public health interventions.

Ultimately, language use in public health interventions is the perfect avenue for interdisciplinary research partnerships between public health and linguistic anthropology. Culturally tailoring education materials is the beginning of a more attuned public health intervention practice [10]. Successful communication of education through interventions requires assessment of communicative practices, how those practices are meaningful to participants, and what role they play in the intervention. These recommendations place a focus on breaking down borders between health providers and educators, disciplines, and unique populations to steer public health interventions toward community-centered interventions—thus designing programs which can lead us toward health/communicative justice [40]. Utilizing strengths from other disciplines and experts to further tailor interventions to better communicate with specific communities could impact study outcomes, program sustainability, community partnerships, knowledge between disciplines, and future research methodologies.

## Data Availability

Data are not available because there are previously agreed upon data sharing agreements with each tribe which prevents making datasets publicly available.
